# RNAi mediated acute depletion of Retinoblastoma protein (pRb) promotes aneuploidy in human primary cells via micronuclei formation

**DOI:** 10.1186/1471-2121-10-79

**Published:** 2009-11-02

**Authors:** Angela Amato, Laura Lentini, Tiziana Schillaci, Flora Iovino, Aldo Di Leonardo

**Affiliations:** 1 Dipartimento di Biologia Cellulare e dello Sviluppo "A. Monroy", Università di Palermo, viale delle Scienze, Palermo, Italy; 2 Dipartimento di Discipline Chirurgiche e Oncologiche, Laboratorio di Patofisiologia Cellulare e Molecolare Università di Palermo, Palermo, Italy

## Abstract

**Background:**

Changes in chromosome number or structure as well as supernumerary centrosomes and multipolar mitoses are commonly observed in human tumors. Thus, centrosome amplification and mitotic checkpoint dysfunctions are believed possible causes of chromosomal instability. The Retinoblastoma tumor suppressor (*RB*) participates in the regulation of synchrony between DNA synthesis and centrosome duplication and it is involved in transcription regulation of some mitotic genes. Primary human fibroblasts were transfected transiently with short interfering RNA (siRNA) specific for human pRb to investigate the effects of pRb acute loss on chromosomal stability.

**Results:**

Acutely pRb-depleted fibroblasts showed altered expression of genes necessary for cell cycle progression, centrosome homeostasis, kinetochore and mitotic checkpoint proteins. Despite altered expression of genes involved in the Spindle Assembly Checkpoint (SAC) the checkpoint seemed to function properly in pRb-depleted fibroblasts. In particular *AURORA-A *and *PLK1 *overexpression suggested that these two genes might have a role in the observed genomic instability. However, when they were post-transcriptionally silenced in pRb-depleted fibroblasts we did not observe reduction in the number of aneuploid cells. This finding suggests that overexpression of these two genes did not contribute to genomic instability triggered by *RB *acute loss although it affected cell proliferation. Acutely pRb-depleted human fibroblasts showed the presence of micronuclei containing whole chromosomes besides the presence of supernumerary centrosomes and aneuploidy.

**Conclusion:**

Here we show for the first time that *RB *acute loss triggers centrosome amplification and aneuploidy in human primary fibroblasts. Altogether, our results suggest that pRb-depleted primary human fibroblasts possess an intact spindle checkpoint and that micronuclei, likely caused by mis-attached kinetochores that in turn trigger chromosome segregation errors, are responsible for aneuploidy in primary human fibroblasts where pRb is acutely depleted.

## Background

Genomic instability is a hallmark of the vast majority of human cancers. The predominant form of genomic instability in human cancer is chromosome instability (CIN), which is characterized by gains or losses of whole chromosomes (aneuploidy) and chromosomal structural aberrations [1]. Recent studies have shown that CIN and aneuploidy, a long time considered late progression events to be associated with tumors, indeed represent early molecular changes seen in preneoplastic lesions of human cancers [2].

Aneuploidy occurrence could generate in a single step multiple changes required for tumor initiation and progression and is frequently observed in clinical tumor specimens. However, it is still debated whether aneuploidy is the consequence or the cause of tumorigenesis [3,4]. Duplicated chromosomes must be equally segregated between the two daughter cells during cell division, and errors in this process can lead to aneuploidy. The presence of chromosomal gains and losses, particularly at early stages of carcinogenesis, has suggested that the impairment of chromosome segregation fidelity might play a central role in the genesis of cancers. However, the mechanism responsible for aneuploidy in the earliest stages of tumorigenesis is poorly understood. At least two possible causes, not mutually exclusive, could be responsible for aneuploidy: mutations in genes encoding mitotic regulators, like spindle assembly checkpoint (SAC) proteins, and defects in centrosome homeostasis.

Altered expression of genes involved in the SAC that monitors the correct alignment and attachment of chromosomes to the mitotic spindle, such as *BUB1*, *PTTG1*, *MAD2 (Mad2L1) *and *BUB1B*, induced aneuploidy in mammalian cells in culture, however, they were rarely found mutated in human tumors [5-7]. Further studies provided evidence that reduced expression of some of these genes contributes to defective spindle checkpoint controls. In fact deletion of one *MAD2 *allele resulted in a defective mitotic checkpoint in both human tumor cells and murine primary fibroblasts (MEFs), and *BUB1B *haploinsufficiency in mice resulted in defective mitotic arrest as well as tumors [8,9]. Recently, it was reported a hereditary mutation of the *BUB1B *gene in patients with mosaic variegated aneuploidy (MVA) a rare genetic disease with increased cancer risk [10]. Furthermore, *MAD2 *overexpression was associated with stable inactivation of the Retinoblastoma (*RB*) gene by specific short hairpin RNAs (shRNAs) [11].

Already in the past century Theodor Boveri (1914) observed that cells with supernumerary centrosomes mis-segregated their chromosomes through the assembly of multipolar spindles. Centrosome amplification (indicating both numeric and morphological alterations) is a frequent event observed in the majority of solid tumors and it has also been detected in leukemia and lymphoma cells [12]. Moreover, centrosome amplification has been associated with genetic instability observed in prostate [13] and breast cancer [2] as well as in preinvasive cancer lesions [14]. Then, the correct centrosome number, necessary to assemble a bipolar mitotic spindle, is essential for chromosome segregation fidelity thus preventing CIN and aneuploidy.

Alterations in the two major tumor suppressors *RB *and *TP53 *have been reported to affect chromosome stability. Overexpression of *CYCLIN-E *and mutation of *hCDC4 *gene have been associated with CIN and centrosome amplification [15], and recently it has been reported that also p53 deficient cells needed *CYCLIN-E *overexpression to induce CIN and multiple centrosomes [16]. Halting mitotic progression in human and murine pRb deficient primary fibroblasts resulted in supernumerary centrosomes and aneuploidy [17]. Inactivation of pRb in human keratinocytes [18] by expression of the HPV16-E7 oncoprotein as well a transient G1/S cell cycle arrest in human fibroblasts and Mouse Embryonic Fibroblasts (MEFs) with pRb dysfunction also generated multiple centrosomes and aneuploidy [19]. Therefore, a tightly controlled coupling of centrosome and DNA replication and the mitotic cycle is necessary to generate correct segregation for both chromosomes and centrosomes.

To avoid any side effects caused by additional gene alterations present in tumor cell lines, we used IMR90 human diploid primary fibroblasts to investigate the mechanism(s) by which cells might become aneuploid. To this aim we analyzed the effects of acute loss of the Retinoblastoma (*RB*) gene function on centrosomes and genomic instability by using the RNA interference (siRNAs mediated) knockdown methodology. Here we show that in addition to supernumerary centrosomes acutely pRb-depleted human fibroblasts harbored micronuclei. Our results indicate that micronuclei formation caused by aneugenic events leading to chromosome loss, likely originating from erroneous kinetochore attachment, and not spindle assembly checkpoint dysfunctions are the trigger for aneuploidy in pRb-depleted primary human fibroblasts.

## Results

### Acute loss of pRb in primary human fibroblasts generates supernumerary centrosomes, aneuploidy and changes in expression of genes necessary for centrosome duplication and mitotic progression

**Figure 1:**
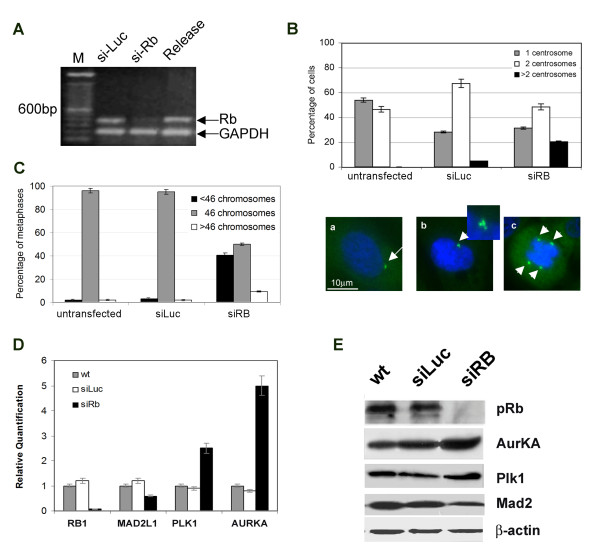
**pRb depletion causes centrosome amplification, aneuploidy and altered gene expression**. **A) **RT-PCR analysis for *RB *expression in IMR90 cells transfected with siRNA-luciferase (siLuc), siRNA targeting *RB *(siRB) and after *RB *re-expression (release). **B) **The histogram (top panel) summarizes the percentage of cells with 1, 2 or >2 centrosomes in control and pRb-depleted IMR90 cells. Presence of supernumerary centrosomes in interphase cells (bottom panel: b) and abnormal mitotic spindles (bottom panel: c, arrowheads) in IMR90 cells transfected with siRNA targeting *RB *in comparison with control cells (bottom panel: a) as revealed by immunofluorescence for γ-tubulin (green) or β-tubulin (green) respectively, nuclei were stained with DAPI (blue). **C) **Bar graphs showing the percentage of cells with normal (46 chromosomes), hypodiploid (<46 chromosomes) and hyperdiploid (>46 chromosomes) metaphases after *RB *silencing and in control cells, untransfected or transfected with siRNA-luciferase (siLuc). **D) **Expression levels of genes involved in mitosis progression by Real time RT-PCR. The x-axis indicates genes and the y-axis the relative quantification in IMR90 cells untransfected (wt), transfected with siRNA-luciferase (siLuc) and siRNA targeting *RB *(siRB). **E) **Western blot analysis in pRb silenced IMR90 cells showing lack of pRb, increase of Aurora-A, Plk1 and decrease of Mad2 protein at 72 hours. β-actin is used a loading control.

Previously, we showed that in pRb deficient human cells (expressing the HPV16-E7 oncoprotein) centrosome amplification triggered aneuploidy [19] and acute loss of pRb in MEFs caused centrosome amplification and aneuploidy [20]. However, because HPV16-E7 oncoprotein could have additional cellular effects besides pRb inactivation, and murine cells have less stringent checkpoints than human cells, we used primary human fibroblasts to investigate whether pRb acute loss by RNA interference affected chromosomal stability. Conventional RT-PCR and Western blotting showed that transfection of siRNA oligonucleotides specifically targeting *RB *reduced both *RB *transcript and protein levels when compared with cells transfected with control siRNA (Figure 1A). Reduction in gene expression was transient as shown by *RB *re-expression after additional 72 hours in culture (Figure 1A: release).

The immediate early effects of pRb transient post-transcriptional silencing (acute loss) on centrosome homeostasis were revealed by detection of the centrosomal component γ-tubulin (Figure 1B). *RB *acute loss generated supernumerary centrosomes (Figure 1B, b) in 20% of cells in comparison to control cells (Figure 1B, a) that showed only 5% of cells with multiple centrosomes. The number of pRb-depleted cells with amplified centrosomes decreased after ten days in culture (data not shown). Supernumerary centrosomes generating multipolar spindle, as revealed after β-tubulin immunostaining, might be responsible for chromosomal missegregation and successive aneuploidy observed after acute loss of pRb in human fibroblasts (Figure 1B, c).

Then we addressed whether these cells had acquired genomic instability by carrying out conventional cytogenetics analyses. Alterations in the normal number of chromosomes were frequently observed in human fibroblasts acutely depleted of pRb but not in their wild-type parental counterpart. Karyotype analysis done after 4 days from siRNAs transfection revealed aneuploidy in 50% of analyzed metaphases (Figure 1C) and the majority (80%) were hypodiploid (< 46 chromosomes). Recently, we found that in pRb deficient murine cells acute loss of pRb induced altered expression of genes involved in centrosome homeostasis and mitosis progression [20]. Then, we determined whether also in primary human fibroblasts acute loss of pRb altered the expression of some genes involved in chromosomal/centrosome dynamics that might be relevant for genomic stability. Quantitative real time RT-PCR confirmed that IMR90 cells at 72 hours from transfection had a very low level of *RB *expression (Figure 1D). We found increased transcripts levels of genes necessary for centrosome duplication and mitotic progression as *AURORA-A *and *PLK1 *(Figure 1D). In addition pRb acute loss was associated with decreased *MAD2 *transcript levels (Figure 1D) a gene belonging to the category of the Spindle Assembly Checkpoint (SAC) genes. Western blotting (Figure 1E) and successive densitometry analysis of Western blots confirmed Real Time RT-PCR results. In particular quantification of Western blot band intensity indicated a two-fold increase for *AURORA-A *protein level, about 1.5 fold change for *PLK1 *protein level and a 50% reduction of MAD2 protein level.

### Evaluation of the spindle assembly checkpoint involvement in triggering chromosomal instability of RB silenced IMR90 cells

**Figure 2:**
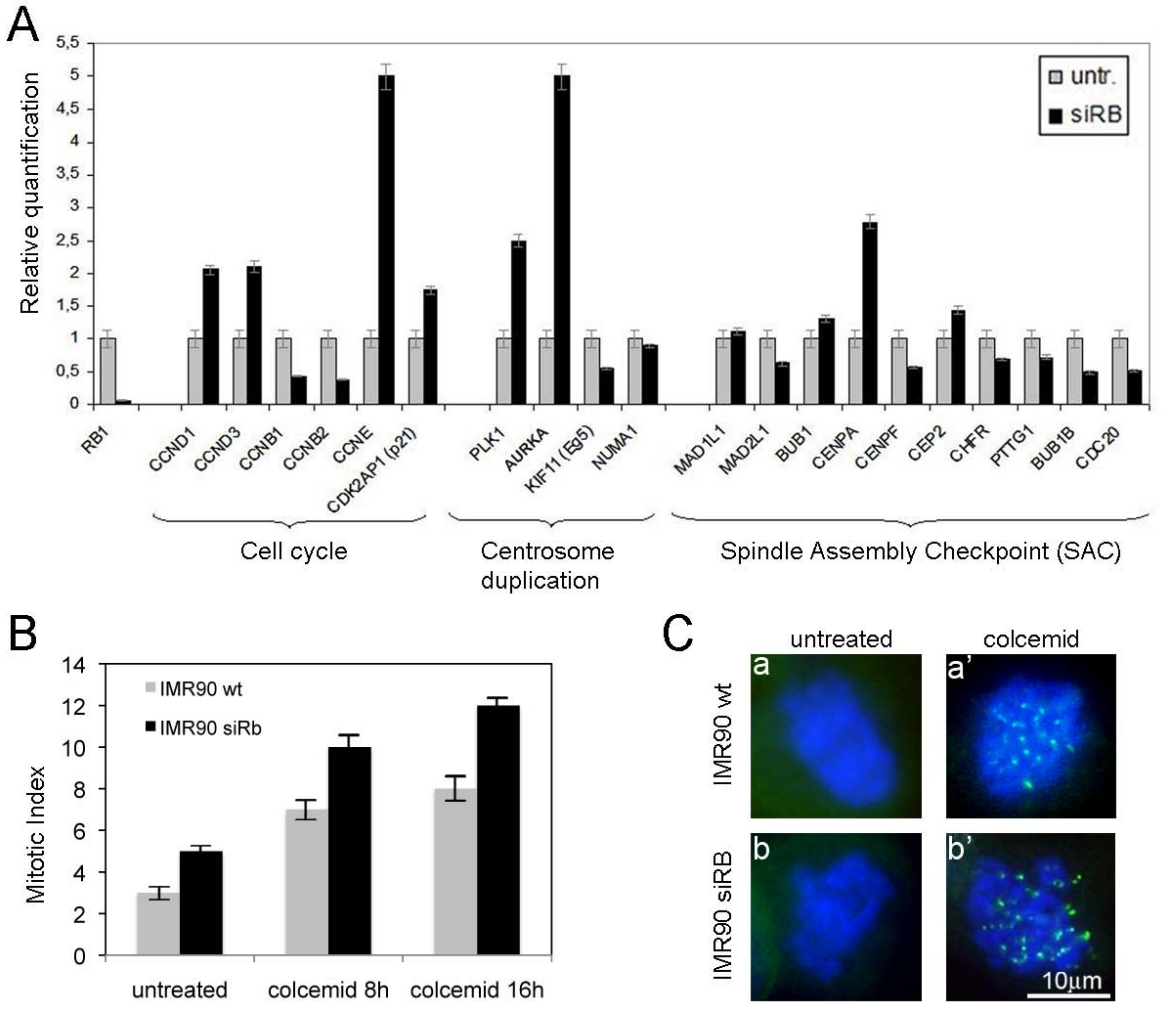
**Evaluation of the spindle assembly checkpoint dysfunction in triggering chromosomal instability of pRb-depleted cells**. **A) **Expression levels of genes involved in cell cycle regulation, centrosome duplication and mitosis progression by TaqMan Low Density Array (TLDA). The x-axis indicates genes and y-axis the relative quantification in IMR90 cells untransfected (untr.) and transfected with siRNA targeting *RB *(siRB). **B) **Bar graphs showing increased mitotic index in untransfected (wt) and pRb-depleted (siRB) IMR90 cells after colcemid treatment (colcemid 8 h and 16 h). **C) **Immunofluorescence analysis detected Mad1 localization (green spots) in both wild-type and pRb-depleted cells after colcemid treatment (colcemid treated, a' and b' respectively) but not in untreated wild-type and pRb-depleted cells (untreated, a and b respectively). Nuclei were counterstained with DAPI (blue).

Several lines of evidence implicate that a weakened Spindle Assembly Checkpoint (SAC) could be responsible for the generation of aneuploid cells [8]. The observed decrease in *MAD2 *expression in transient *RB *silenced IMR90 cells (Figure 1D), resulting in the reduction of a critical SAC component, might be consistent with this view. To determine if other mitotic checkpoint genes in addition to *MAD2 *were also altered in transient *RB *silenced IMR90 cells we extended the Real time RT-PCR to other genes involved in cell cycle, centrosome duplication and Spindle Assembly Checkpoint. Our analysis showed a marked decrease in the expression of *BUB1B *and *CDC20 *(about 50% of reduction), *PTTG1 *and *CHFR *(about 30% of reduction) in pRb acutely depleted cells in comparison to control cells (Figure 2A). In addition we detected differential expression of genes coding for centromeric proteins namely centromere protein-A (*CENP-A*) overexpression and centromere protein-F (*CENP-F*) down-regulation. Altogether, the reduced expression levels of these genes suggested a possible SAC dysfunction following pRb acute loss. To this aim we estimated the number of mitotic cells (mitotic index) in pRb-depleted cells after treatment with the mitotic inhibitor colcemid that depolymerizes microtubules and blocks metaphase-anaphase transition by activating the SAC. Our data indicated that both pRb-competent and pRb-depleted cells arrested in mitosis exhibiting an increased mitotic index (Figure 2B) [see Additional file 1]. Furthermore, to determine that pRb-depleted cells arrested because of SAC activation we looked at kinetochore accumulation of Mad1, another crucial SAC component, after challenging the checkpoint with colcemid. To this end we looked at Mad1 localization of both pRb-competent and pRb-depleted IMR90 cells, treated with colcemid or left untreated, by immunofluorescence microscopy. As expected after colcemid treatment Mad1 accumulated at kinetochores of both pRb-competent and pRb-depleted cells (Figure 2C a', and 2b'). On the contrary no Mad1 signals were detected in both pRb-competent and pRb-depleted untreated cells (Figure 2C, a and 2b). These results indicate that human fibroblasts acutely depleted of pRb possess a functional SAC, suggesting that pRb-depleted cells did not undergo a cell cycle arrest before reaching mitosis as revealed by the decrease in gene expression of mitotic genes we observed by Real time RT-PCR (Fig 2A). Therefore, previous findings that tumor cells with *MAD2 *haploinsufficiency did not arrest in mitosis and proceed into a subsequent S phase after mitotic poison treatment [8], are likely to be attributed to some additional genetic mutations present in these tumor cell lines tested.

### Genome instability caused by pRb acute loss depends neither on PLK1 nor on AURORA-A overexpression

**Figure 3:**
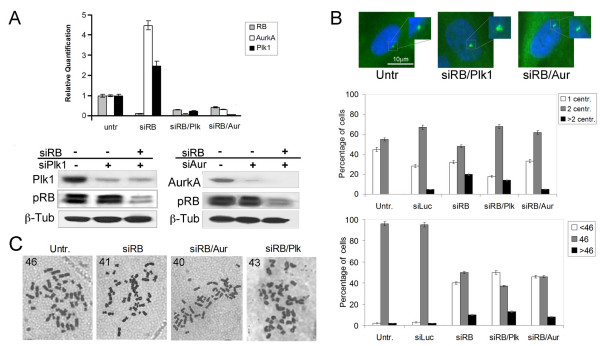
**Effects of simultaneous *RB/AURORA-A *and *RB/PLK1 *silencing in IMR90 cells**. **A)** Gene expression levels of *RB*, *AURORA-A *and *PLK1 *(top panel) evaluated by Real Time RT-PCR in silenced IMR90 cells as indicated. Western blots detecting protein levels for RB, Aurora-A (right bottom panel) and for RB, Plk1 (left bottom panel) in silenced IMR90 cells as indicated; β-tubulin was used as a loading control.**B) **Immunofluorescence microscopy detecting centrosome numbers (top panel) in untreated and double knockdown IMR90 cells. The graph in the middle panel summarizes the percentage of IMR90 cells with 1, 2 or more than 2 centrosomes after RNAi of *RB*, *RB/AURORA-A *and *RB/PLK1*. In the bottom panel are shown histograms summarizing hypodiploid (<46 chromosomes), diploid (46 chromosomes) and hyperdiploid (>46 chromosomes) metaphases. Cytogenetics analysis was carried out after three days following transfection except in *RB/AURORA-A *transcriptionally silenced fibroblasts which were examined after four days. **C)** Examples of metaphase spreads fro the indicated cell types. Numbers refer to chromosomes.

**Figure 4:**
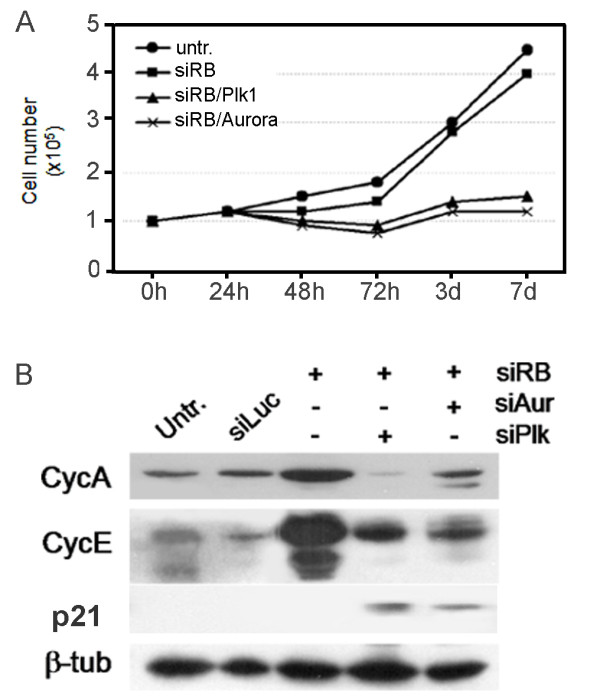
**Evaluation of cell growth at different times after *RB *silencing**. **A) **Decreased proliferation rates starting 48 h up to 7 days from transfection in *RB/PLK1 *and *RB/AURORA-A *co-depleted cells (double knock-down) in comparison with both untransfected and pRb-depleted cells. **B) **Western blot analysis showing increased Cyclin-E protein levels in pRb-depleted cells as well as in cells with knock-down of both *RB *and *AURORA-A *or *PLK1*, and increased Cyclin-A protein levels in *RB/AURORA-A *co-depleted cells. Cells with knock-down of both *RB *and *AURORA-A *or *PLK1 *but not with *RB *knock-down showed increased p21^Cip1 ^levels. β-tubulin was used as a loading control of protein extracts.

To investigate the role of *AURORA*-*A *and *PLK1 *overexpression on centrosome amplification and genomic instability we silenced them by RNA interference (RNAi) in acutely pRb-depleted cells. Real time RT-PCR showed reduced levels of *RB*, as well as *AURORA-A *and *PLK1 *transcripts, in IMR90 cells subjected to knock-down of both *RB *and *AURORA-A *or *PLK1 *(Figure 3A, top panel). Western blot analysis confirmed protein decrease in post-transcriptionally silenced cells (Figure 3A, bottom panels). By immunofluorescence microscopy (Figure 3B, top panel) we observed that cells with multiple centrosomes were slightly reduced after simultaneous RNAi of *RB *and *PLK1 *(14%) in comparison to *RB *alone post-transcriptionally silenced cells (18%). In contrast post-transcriptional *RB *and *AURORA-A *silencing caused a marked reduction of supernumerary centrosomes in *RB/AURORA-A *post-transcriptionally silenced cells (Figure 3B, middle panel). These findings suggest that centrosome amplification observed in *RB *depleted fibroblasts as well in *RB/PLK1 *depleted cells might depend on *AURORA-A *overexpression. However, real time RT-PCR done after *RB/PLK1 *double knockdown revealed a very low level of *AURORA-A *mRNA (Figure 3A) suggesting that in these cells centrosome amplification did not depend, at least in part, on *AURORA-A *overexpression. Next, we did conventional cytogenetics to evaluate the presence of aneuploidy in *RB/PLK1 *and *RB/AURORA-A *post-transcriptionally silenced fibroblasts. We scored an increase in hypodiploid cells both in *RB/PLK1 *and *RB/AURORA-A *post-transcriptionally silenced fibroblasts (Figure 3B, bottom panel). These findings suggest that pRb acute loss could promote genomic instability that depends neither on *PLK1 *nor on *AURORA-A *overexpression. Alternatively, both up-regulation and down-regulation of these two genes could affect genomic stability in primary pRb-depleted cells. In addition the presence of supernumerary centrosomes did not seem the only cause leading to genomic instability in *RB *post-transcriptionally silenced cells. In the attempt to establish a possible role for the observed *PLK1 *or *AURORA-A *overexpression in *RB *post-transcriptionally silenced cells, we looked at proliferation of these cells in culture. Following simultaneous RNA interference of *RB/PLK1 *and *RB/AURORA-A*, IMR90 cells showed a delay in cell proliferation already at 72 hours, and at ten days from transfection these post-transcriptionally silenced cells underwent a permanent arrest. On the contrary pRb alone depleted cells recovered from the initial proliferation delay as soon as two days from transfection (Figure 4A). Moreover, Western blot analyses showed increased levels of p21^Cip1 ^in cells with knock-down of both *RB *and *AURORA-A or PLK1 *but not in *RB *alone depleted cells, suggesting a possible arrest of proliferation in a small fraction of cells co-depleted for *RB *and either *PLK1 *or *AURORA-A*. The decreased amounts of Cyclin-E and Cyclin-A observed in *RB/PLK1 *and *RB/AURORA-A *double knocked-down IMR90 cells in comparison with the *RB *single knocked-down IMR90 cells are consistent with a proliferation delay of these cells (Figure 4B).

### Micronuclei generation occurred following Rb acute loss in IMR90 human primary fibroblasts

**Figure 5:**
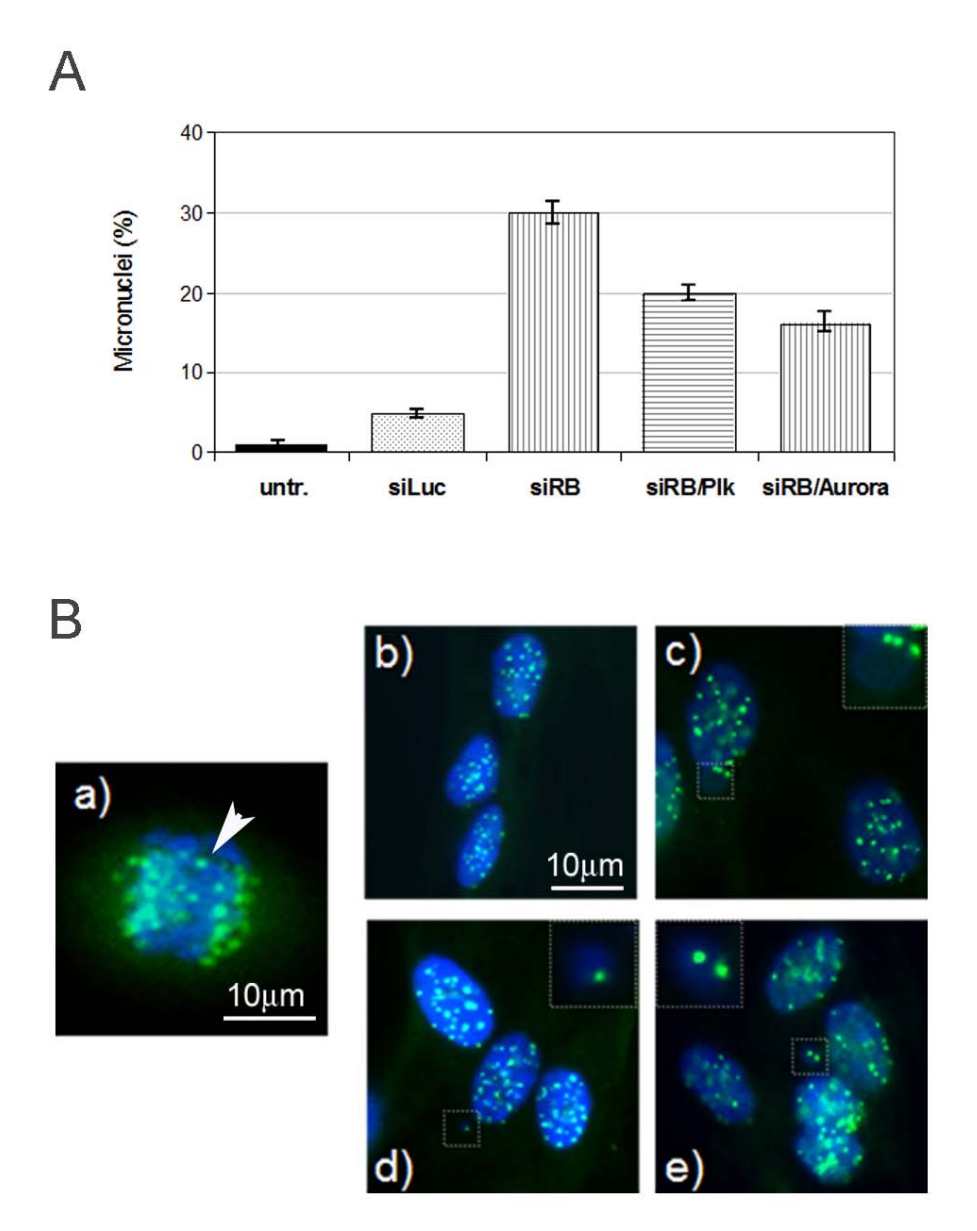
**Micronuclei generation after pRb acute loss in primary human fibroblasts**. **A) **Bar graphs showing micronuclei percentages scored in *RB *transcriptionally silenced IMR90 cells (siRB) and in IMR90 cells with knock-down of both *RB *and *AURORA-A *or *PLK1 *compared with untransfected cells (untr.) or cells transfected with siRNA-luciferase (siLuc). **B) **Immunofluorescence microscopy analysis detecting the presence of centromeres (green spots) in IMR90 wild-type cells (b), in micronuclei generated following *RB *transcriptional silencing (c), and after knock-down of both *RB *and *PLK1 *(d) or after knock-down of both *RB *and *AURORA-A *(e). In (a) are shown lagging chromosomes (arrow) observed during metaphases in RB transcriptionally silenced cells. Nuclei were stained with DAPI (blue).

To investigate a possible mechanism by which *RB *acute loss participates in the generation of aneuploid cells we did cytogenetics analysis looking for the presence of micronuclei. Micronuclei are considered a marker of chromosome damage (chromosome loss or chromosome breakage events) formed during mitosis and identified in interphase as small bodies of extra chromatin in the cytoplasm of mammalian cells. We found the presence of micronuclei in pRb-depleted cells (30%) as well as in both *RB/PLK1 *(20%) and *RB/AURORA-A *(18%) double knockdown cells (Figure 5A). To determine whether micronuclei harbored acentric chromosomal fragments or whole chromosomes we investigated the presence of centromeres in these micronuclei. Immunofluorescence microscopy conducted using an anti-centromere antibody (CREST) showed that micronuclei contained centromeres suggesting the presence of whole chromosomes therein (Figure 5B c, d, e). The control wild type cells did not show the presence of micronuclei by fluorescence microscopy (Figure 5B, b). In addition immunofluorescence microscopy showed the presence of more than one centromeric signal in micronuclei of *RB *silenced cells suggesting the loss of more than one chromosome per cell, that is consistent with the hypodiploid phenotype displayed by pRb-depleted cells. Micronuclei might originate by the presence of lagging chromosomes observed during anaphase in human fibroblasts after *RB *acute loss (Figure 5B-a). This result suggests that micronuclei, which remain separate from the nucleus after nuclear division, might be the most likely way by which pRb-depleted cells become hypodiploid.

## Discussion

Chromosomal instability plays an important role in the initiation, progression and aggressiveness of cancer cells. Unlike point mutations that affect only some genes, gains or losses of chromosomes (i.e., aneuploidy) altering dramatically gene expression could generate in a single step multiple changes required for tumor initiation and progression. An aneuploid cell will usually have a higher number of chromosomes (sometimes lower) and will not divide properly, resulting in two cells with different numbers of chromosomes. The mechanisms implicated in the formation of aneuploid cells are still not fully understood. Both alterations in genes controlling centrosome homeostasis and defects in genes encoding mitotic checkpoint proteins could trigger aneuploidy. The presence of supernumerary centrosomes is considered a possible cause of both chromosomal instability and aneuploidy. Supernumerary centrosomes have been associated with p53 and pRb dysfunction caused by the presence of *HPV16-E6 *and -*E7 *oncoproteins [21]. However, we showed that pRb but not p53 deficient human cells resulted in aneuploid cells by allowing DNA re-replication associated with centrosomes amplification [17].

In mammalian cells DNA replication and centrosome duplication must be completed successfully before mitosis and are tightly regulated by a control network in which pRb plays a crucial role. Previously, we showed that centrosome amplification and aneuploidy occurred only after a prolonged G1/S arrest in MEFs stably devoid of pRb. In addition the presence of these alterations in pRb acutely depleted MEFs highlighted the importance of pRb in the control of genetic stability [20].

Here we demonstrate for the first time that *RB *acute loss triggers centrosome amplification and aneuploidy in human primary fibroblasts. We observed reduced expression of crucial Spindle Assembly Checkpoint (SAC) components suggesting that this checkpoint might be weakened in these cells and in turn it might be responsible for the observed aneuploidy. However, though fibroblasts acutely depleted of pRb showed *MAD2 *reduction, they did not show premature sister chromatid separation (PCS) that is a hallmark of tumor cells with reduced levels of *MAD2 *and mitotic checkpoint dysfunction [8]. In addition when pRb acutely depleted IMR90 cells were treated with the mitotic poison colcemid they accumulated in mitosis indicating activation and functionality of the SAC checkpoint. Altogether these findings suggest that aneuploidy might not be triggered by SAC dysfunction in human primary fibroblasts acutely depleted of pRb.

Recently, it was reported that *MAD2 *up-regulation because of stable pRb depletion by short hairpin RNAs (shRNAs) led to chromosomal instability in human cells [11]. The explanation for this effect of overexpressed *MAD2 *was that part of the population adapted to the activated checkpoint and entered the cell cycle as polyploid cells that in turn could cause chromosome missegregation in the subsequent mitoses. On the contrary our results indicated that acute loss of *RB *in normal human fibroblasts did not cause *MAD2 *overexpression. Our findings suggest that the previously observed *MAD2 *overexpression could be a specific feature of cells stably depleted of pRb. Indeed, we found that *MAD2 *was overexpressed in IMR90 human fibroblasts stably depleted of pRb by transfection of a shRNA-mir (RB.670) targeting specifically human *RB *(data not shown). We found *MAD2 *up-regulation also in pRb-depleted HCT116 cells (A.A. manuscript in preparation), where this up-regulation likely depended on overexpression of *BRCA1 *that binds to the transcription factor OCT-1 and up-regulates *MAD2 *transcription [22]. Thus, additional genetic changes that occurred during the selection of *RB *stably depleted cells might be the explanation for up-regulation and to be involved in overriding the mitotic checkpoint if activated.

Critical regulators of mitotic progression and centrosome duplication such as *PLK1 *and *AURORA-A *that are expressed in a cell cycle dependent manner were also upregulated after RB acute loss. Recently, it has been shown that activation of the RB pathway resulted in the repression of Plk1 promoter activity that depended on the SWI/SNF chromatin-remodeling complex [23]. Aurora-A overexpression following pRb depletion is consistent with recent finding that this kinase is under the transcriptional control of E2F3 [24], a partner of pRb in the regulation of cell cycle transitions. Also Plk1 has been reported to be regulated by E2F3 in bladder cancer cells in addition to known E2F3 targets such as Cyclin A and novel targets including pituitary tumor transforming gene 1 (PTTG1), and Caveolin-2 [25]. Very recently, several findings have outlined the existence of the functional crosstalk between Plk1 and Aurora-A. Plk1 is activated before mitosis by Aurora-A and its cofactor Bora [26]. Furthermore, in mitosis Bora degradation is Plk1 dependent, and Plk1 is also required for the timely destruction of Aurora-A in late anaphase [27]. To understand the roles played by *PLK1 *and *AURORA-A *overexpression on centrosome amplification and aneuploidy, following pRb acute depletion, we did post-transcriptional silencing of *RB*, *PLK1 *and *RB*, *AURORA-A *simultaneously. Only *RB/AURORA-A *silenced human fibroblasts showed a decrease in the percentage of cells with supernumerary centrosomes suggesting that *AURORA-A *over-expression might be necessary for centrosome amplification to occur. However, *RB/PLK1 *silenced cells showed low transcript levels for *AURORA-A *suggesting that *AURORA-A *overexpression could be in part responsible for centrosome amplification.

On the other hand conventional cytogenetics analysis showed the presence of aneuploidy (mainly hypodiploid metaphases) in both *RB/AURORA-A *and *RB/PLK1 *silenced fibroblasts, suggesting that neither *AURORA-A *nor *PLK1 *over-expression could account for chromosome instability observed in these pRb-depleted cells. In this scenario it seems that *AURORA-A *and *PLK1 *over-expression affects primarily cell proliferation following pRb depletion in human primary fibroblasts. In fact simultaneous post-transcriptional silencing of *RB/AURORA-A *and *RB/PLK1 *resulted in delayed cell cycle progression consistent with the decreased Cyclin -E and -A levels, the reduced mitotic index as well as the increased p21^Cip1 ^level. Surprisingly, we observed that aneuploid cells induced by pRb depletion were mostly hypodiploid. The unexpected finding that aneuploidy in pRb-depleted human normal cells is characterized by a unbalanced number of cells with chromosome loss relative to gain, is consistent with what has been recently reported for CENP-E^+/- ^mice, where hypodiploid cells are better tolerated than hyperdiploid cells in vivo [28]. A molecular mechanism underlying this phenotype might involve lack of attachment or erroneous kinetochores attachment to the mitotic spindle. Merotelic kinetochore attachment that occurs when a kinetochore binds mitotic microtubules from both spindle poles could explain hypodiploidy generation [29,30]. Chromosomes with merotelic orientation that are not detected by the SAC because they do not expose unattached centromeres, could result in lagging chromosomes that persisting until anaphase onset would originate micronuclei upon nuclear envelope reconstitution at the end of mitosis [29]. Micronuclei are considered a possible cause of chromosome loss during mitosis resulting in the generation of hypodiploid cells that lack few chromosomes. Indeed immunofluorescence microscopy performed in pRb-depleted cells and in *RB/AURORA-A *and *RB/PLK1 *knockdown cells showed the presence of micronuclei containing whole chromosomes. An alternative hypothesis might be that micronuclei originate in *RB *silenced cells by defects in kinetochore assembling that generates dysfunctional centromere unable to contact correctly mitotic microtubules. Then, altered expression and dosage of some centromere components by promoting defects in kinetochore assembling could trigger micronuclei generation resulting in unfaithful chromosome segregation during mitosis. Expression studies have suggested a correlation between altered expression of several centromere proteins and cancer. Accordingly, in acutely pRb-depleted human primary fibroblasts we found altered expression of genes coding for two centromere proteins: namely *CENP-A *that was overexpressed and *CENP-F *that was underexpressed. *CENP-A *overexpression might cause a spreading of centromere heterochromatin which might interfere with the correct kinetochore complex assembling and then cause CIN as reported in colorectal cancer tissues [31,32]. *CENP-F *(mitosin) associates preferentially with kinetochores of unaligned chromosomes and is degraded upon completion of mitosis, both its overexpression and underexpression has been reported in cancer [33,34]. Altogether, our results suggest that pRb plays a critical role in regulating chromosome stability in human primary fibroblasts and its deficiency triggers defects in chromosome segregation via micronuclei formation. In fact pRb depleted primary human fibroblasts did not show SAC dysfunction despite decreased expression of some SAC genes. Thus, micronuclei could represent a mechanism underlying chromosome instability following *RB *acute loss and they could explain maintenance of hypodiploidy also after that *RB *expression will be restored.

## Conclusion

Here we show for the first time that *RB *acute loss triggers centrosome amplification and aneuploidy in human primary fibroblasts. Even though pRb acutely depleted human fibroblasts showed *MAD2 *reduction they did not show spindle assembly checkpoint (SAC) dysfunction when challenged with a mitotic poison.

Our observation that pRb-depleted cells were mostly hypodiploid suggests that micronuclei, likely caused by mis-attached kinetochores which in turn triggered chromosome segregation errors, are a possible cause of chromosome loss during mitosis resulting in the generation of aneuploid cells that lack few chromosomes. Altogether, our results suggest that pRb plays a critical role in regulating genomic stability in human primary fibroblasts and its deficiency induces defects in chromosome segregation via micronuclei formation and not by SAC dysfunction.

## Methods

### Cells and Cell Culture

Normal human IMR90 fibroblasts (diploid human embryonic lung fibroblasts, ATCC CCL-186) were cultured in MEM (Minimum Essential Medium) supplemented with 10% FBS (Gibco, EU approved, Invitrogen Italy), 2 mM L-glutamine, 100 U/ml penicillin, 0,1 mg/ml Streptomycin, 1 mM Sodium Pyruvate and Non Essential Amino Acid (Invitrogen, Italy). For siRNAs transfection 1 × 10^5 ^cells were plated in 6-well plates and incubated overnight at 37°C. Specific siRNAs duplexes were mixed with Lipofectamine2000 Reagent according to manufacturer's recommendation and added to the cells. After 16 h at 37°C, the medium was replaced. For *RB *silencing we used a combination of four siRNAs (80 nM siRB smart pool Dharmacon Inc) recognizing four specific regions of the *RB *transcript, for Aurora-A (siAurora, 60 nM) and Plk1 (siPlk, 60 nM) silencing we used custom single siRNA (Eurofins MWG Operon, Germany). A siRNA duplex targeting the luciferase gene (siLuc, 60 nM, Eurofins MWG Operon, Germany) was used as a control. Analysis of cell cultures was performed after 72 hours from transfection. For proliferation assay cells were plated on 6-well plates in duplicate and at every end point were harvested and stained with Trypan blue solution.

### Western blot analysis

Cells were lysed in SDS/PAGE sample buffer, protein extracts were resuspended in loading buffer (0.125 M Tris-HCl, 4% SDS, 20% v/v Glycerol, 0.2 M dithiothreitol, 0.02% Bromophenol Blue, pH 6.8) and 50 μg of protein (as determined by the Bradford assay) were loaded per lane on a SDS PAGE gel. After gel electrophoresis proteins were electrotransferred onto Immobilon-PVDF membrane (Millipore, Italy) blocked in 5% (w/v) no-fat milk in TBST buffer (10 mM Tris pH8.0,150 mM NaCl, 0.1% Tween 20) at room temperature and incubated overnight at 4°C with the primary antibody. After three washes with TBST buffer the blot was incubated in horseradish peroxidase-conjugated secondary antibody (Santa Cruz Biotechnology, Inc., diluted 1:2000) for 1 h RT. Western blot was probed with mouse monoclonal anti pRB (554136, Becton Dickinson), anti Plk1 (sc-17783 F-8, Santa Cruz) or anti Cyclin-E (SC-247he12 Santa Cruz Biotechnology, Inc.) antibodies. Goat polyclonal Aurora A and Mad2 (sc-14318 N-20 and sc-6329 C-19 respectively, Santa Cruz Biotechnology, Inc.) antibodies. Anti-rabbit Cyclin-A (sc-751h-432 Santa Cruz Biotechnology, Inc.). Polyclonal rabbit anti p21 (sc-756 h-164) antibody. Equal loading of proteins was evaluated by probing the blot with a mouse monoclonal antibody β-actin (A53-16, Sigma-Aldrich, Italy) or β-tubulin (T40-26, Sigma-Aldrich, Italy). Blots were developed with chemiluminescent reagent (Super Signal West Pico, Pierce Rockford, IL) and exposed to CL-Xposure film (Pierce Rockford, IL) for 1 to 5 min. To quantitate the intensity of Western blot bands was used the Image-J software (NIH) available at .

### Cytogenetics analysis

Three days after transfection 3.5 × 10^4 ^cells were seeded on rounded glass coverslips and incubated for 8 h and 16 h with 0,2 μg/ml of Colcemid (Demecolcine Sigma-Aldrich, Italy). To harvest mitotic cells prewarmed hypotonic buffer (75 mM KCl) was dropped onto the coverslips and cell were allowed to swell for 10-15 min at 37°C. Then cell were fixed by adding a methanol/acetic acid (v/v) 3:1 ice-cold solution. The slides were air-dried and stained with 3% Giemsa stain in phosphate-buffered saline for 10 min. Chromosome numbers were evaluated using a Zeiss Axioskop microscope under a 100× objective. At least 50 metaphases were analyzed at each time point.

### Immunofluorescence microscopy

#### γ-tubulin detection

To detect centrosomes, 3.5 × 10^4 ^cells were grown on glass coverslips, fixed in methanol at -20°C, permeabilized with 0.1% Triton X (Sigma-Aldrich, Italy) and blocked with 0.1% BSA both at room temperature. Then, coverslips were incubated with a mouse monoclonal antibody against γ-tubulin (Sigma-Aldrich, Italy, diluted 1:250 in PBS-BSA 0,1%) overnight at 4°C, washed in PBS and incubated with a FITC-conjugated goat anti-mouse (Sigma-Aldrich, Italy, diluted 1:100 in PBS-BSA 0,1%) for 1 h at 37°C. Nuclei were visualized with 4', 6-Diamidino-2-phenylindole (DAPI) and examined on a Zeiss Axioskop microscope equipped for fluorescence. Images were captured with a CCD digital camera (Axiocam, Zeiss, Germany) and then transferred to Adobe PhotoShop for printing.

#### Mad1 immunolocalization

Wild type cells and pRb-depleted cells were grown in duplicate on glass coverslips and incubated with 0,2 μg/ml Colcemid (Demecolcine, Sigma-Aldrich, Italy) for 8 h. Control cells were left untreated. Then cells were fixed in 3.7% formaldehyde solution for 20 min a 37°C, permeabilized with 0.1% Triton X (Sigma-Aldrich, Italy) and blocked with 0.1% BSA, both at room temperature. Coverslips were incubated with a mouse monoclonal antibody against Mad1 (diluted 1:20 in PBS-BSA 0,1%, a kind gift of Dr. A. Musacchio) overnight at 4°C, washed in PBS and incubated with a FITC-conjugated goat anti-mouse (Sigma-Aldrich, Italy, diluted 1:100 in PBS-BSA 0,1%) for 1 h at 37°C. Nuclei were visualized with 4',6-Diamidino-2-phenylindole (DAPI) and examined on a Zeiss Axioskop microscope equipped for fluorescence. Images were captured with a CCD digital camera (Axiocam, Zeiss, Germany) and then transferred to Adobe PhotoShop for printing.

#### Mitotic Index Evaluation

For mitotic index evaluation IMR90 cells both wild-type and RB depleted were grown on glass coverslips. One coverslip per cell type was incubated with 0,2 μg/ml Colcemid (Demecolcine Sigma-Aldrich, Italy) for 8 hours and then fixed with methanol at -20°C, permeabilized with 0.1% Triton X (Sigma-Aldrich, Italy) and blocked with 0.1% BSA, both at room temperature. Then, coverslips were incubated with a mpm2 mouse monoclonal antibody (5 mg/ml in PBS-BSA 0.1%, Upstate) overnight at 4°C, washed in PBS and incubated with a FITC-conjugated goat anti-mouse (Sigma-Aldrich, Italy, diluted 1:100 in PBS-BSA 0,1%) for 1 h at 37°C. Nuclei were visualized with 4',6-Diamidino-2-phenylindole (DAPI) and examined on a Zeiss Axioskop microscope equipped for fluorescence. Images were captured with a CCD digital camera (Axiocam, Zeiss, Germany) and then transferred to Adobe PhotoShop for printing.

#### Centromere detection

To detect centromeres, 3.5 × 10^4 ^cells were grown on glass coverslips, fixed 5 min in methanol/DMEM 1:1 (v/v) and then 30 min in methanol 100% on ice. Coverslips were washed with wash solution (0,1% BSA in PBS 1×) at room temperature and then incubated with a human anti-nuclear antibody overnight at 4°C (Antibodies Inc, Davis, USA), washed and incubated with a FITC-conjugated anti-human antibody (Antibodies Inc, Davis, USA), for 1 h at 37°C. Nuclei were counterstained with 4',6-Diamidino-2-phenylindole (DAPI) and examined on a Zeiss Axioskop microscope equipped for fluorescence. Images were captured with a CCD digital camera (Axiocam, Zeiss, germany) and then transferred to Adobe PhotoShop for printing.

### RNA preparation and RT-PCR

Total RNA was extracted from IMR90 wild type cells, RB knock-down or RB/PLK1 and RB/Aurora-A double knock-down cells by RNAeasy Mini kit according to the manufacture's instruction (Qiagen, Italy).

Total RNA (200 ng) was used for reverse transcriptase reaction (RT) which was carried out using the One step RT-PCR kit (Qiagen, Italy), RT was performed for 30 min at 50°C and 15 min at 95°C. The Amplification cycle (94°C for 45 sec., 55°C for 45 sec, and 72°C for 1 min) was repeated 30 times. PCR primers for RB and GAPDH were designed to produce a DNA fragment of 440 bp or 330 bp in length, respectively. The DNA sequences of primers were: RB 5'-CAGGGTTGTGAAATTGGATCA-3' and 5'-GGTCCTTCTCGGTCCTTTGATTGTT-3'; GAPDH 5'-TGACATCAAGAAGGTGGTGA-3' and 5'-TCCACCACCCTGTTGCTGTA-3'.

#### Real-Time RT-PCR

A subset of genes was selected for quantitative RT-PCR. Primers were designed with Primer Express software (Applied Biosystems, Italy) chosing amplicons of approximately 70-100 bp crossing an exon/exon boundary to minimize the chance that a signal was from contaminating DNA. Total RNA of each sample was retro-transcribed in cDNA by High Capacity cDNA Archive kit (Applied Biosystem, Italy) for 10 minutes at 25°C and 2 h at 37°C. Two microliters (60 ng) of cDNA produced from reverse transcription reactions was added to 12.5 μl of SYBR green 2× PCR Master Mix (Applied Biosystems, Italy), 1.88 μl of 2 mM specific primer pair, and dH2O up to 25 μl final volume. A 7300 Real-Time PCR systems was used to perform real-time fluorescence detection during PCR made up of a step for AmpliTaq Gold Enzyme activation at 95°C for 10 min followed by 40 cycles of PCR (95°C for 15 sec and 60°C for 1 min). Data were analyzed by averaging triplicates C_t _(cycle threshold). Glyceraldehyde-3-phosphate dehydrogenase (GAPDH) was used as internal control. Following the PCR reaction, a melting curve assay was performed to determine the purity of the amplified product.

The sequences of primer pairs used were:

GAPDH: 5'-CTCATGACCACAGTCCATGCC-3' and 5'-GCCATCCACAGTCTTCTGGGT-3',

AURKA: 5'-TTTTGTAGGTCTCTTGGTATGTG-3' and 5'GCTGGAGAGCTTAAAATTGCAG-3',

PLK1: 5'-CGGAAATATTTAAGGAGGGTGA-3' and 5'-GGACTATTCGGACAAGTACG-3',

MAD2L1: 5'-CTCATTCGGCATCAACAGCA-3' and 5'-TCAGATGGATATATGCCACGCT-3',

RB: 5'-GCAGTATGCTTCCACCAGGC-3' and 5'-AAGGGCTTCGAGGAATGTGAG-3'

BRCA1: 5'-CCTTGGCACAGGTGTCCAC-3' and 5'-GCCATTGTCCTCTGTCCAGG-3',

TP53: 5'-TTCGACATAGTGTGGTGGTGC-3' and AGTCAGAGCCAACCTCAGGC-3',

PTTG1: 5'-CGGCTGTTAAGACCTGCAATAATC-3' and 5'-TTCAGCCCATCCTTAGCAACC-3'

CDC20: 5'-GAGGGTGGCTGGGTTCCTCT-3' and 5'-CAGATGCGAATGTGTCGATCA-3',

CHFR: 5'-CCTCAACAACCTCGTGGAAGCATAC-3' and 5'-TCCTGGCATCCATACTTTGCACATC-3'

BUB1B: 5'-TACACTGGAAATGACCCTCTGGAT-3' and 5'-TATAATATCGTTTTTCTCCTTGTAGTGCT-3'

#### TaqMan Low Density Array (TLDA)

TaqMan lowdensity arrays were done according to the manufacturer's instructions using the Applied Biosystems 7900HT fast real-time PCR system (Applied Biosystems, Italy). Total RNA was isolated, measured by photometry and retrotranscripted in cDNA as previously described. A stock solution of 50 ng/ml cDNA was prepared.

Each cDNA sample (10 ml) was added to 2× TaqMan Universal PCR Master Mix (Applied Biosystems) and dH2O up to 500 ml. After gentle mixing and centrifugation, the mixture was then transferred into a loading port on a TLDA card (Applied Biosystems, Italy). The array was centrifuged twice for 1 minute each at 1200 rpm (306 × g) to distribute the samples from the loading port into each well. The card was then sealed and PCR amplification was performed using an Applied Biosystems Prism 7900HT sequence detection system. Thermal cycler conditions were as follows: 2 min at 50°C, 10 min at 95°C, 30 sec at 97°C, 1 min at 60°C for 40 cycles. Each sample was run in duplicate. Results were collected and analyzed by an ABI Prism 7900 Sequence Detection System (PE Applied Biosystems, Italy). Relative RNA levels were calculated using ΔΔC_t _value. In the TaqMan low-density arrays the expression of the target genes was standardized for the expression of quadruplicate housekeeping gene, Glyceraldehyde-3-Phosphate Dehydrogenase (GAPDH, Hs4342376). The reference was the untransfected IMR90 cell line.

## Authors' contributions

AA performed siRNA experiments, Real time RT-PCR, immunofluorescence microscopy, Western blotting, participated in the design of the study and drafted the manuscript. LL carried out RNA preparation for Real time RT-PCR, flow cytometry and helped to draft the manuscript. FI performed siRNA experiments, Real time RT-PCR Western blotting. TS did cytogenetics and proliferation assay and helped to draft the manuscript. ADL conceived of the study, participated in its design and coordination, and wrote the manuscript. All authors read and approved the final manuscript.

## Supplementary Material

Additional file 1 **FACScan analysis of colcemid treated human fibroblasts**. Flow citometry analysis showed increase in G2/M of the cells treated with colcemid up to 16 hours. Cell cycle distribution was determined by using FACScanto (Becton Dickinson) and analyzed by ModFit software (Verity). Click here for file
