# Supplementary figures and images for: RNAi mediated acute depletion of Retinoblastoma protein (pRb) promotes aneuploidy in human primary cells via micronuclei formation
Source: BMC Cell Biol. 2009 Nov 2;10:79. doi: 10.1186/1471-2121-10-79 (PMC2775725; doi:10.1186/1471-2121-10-79)

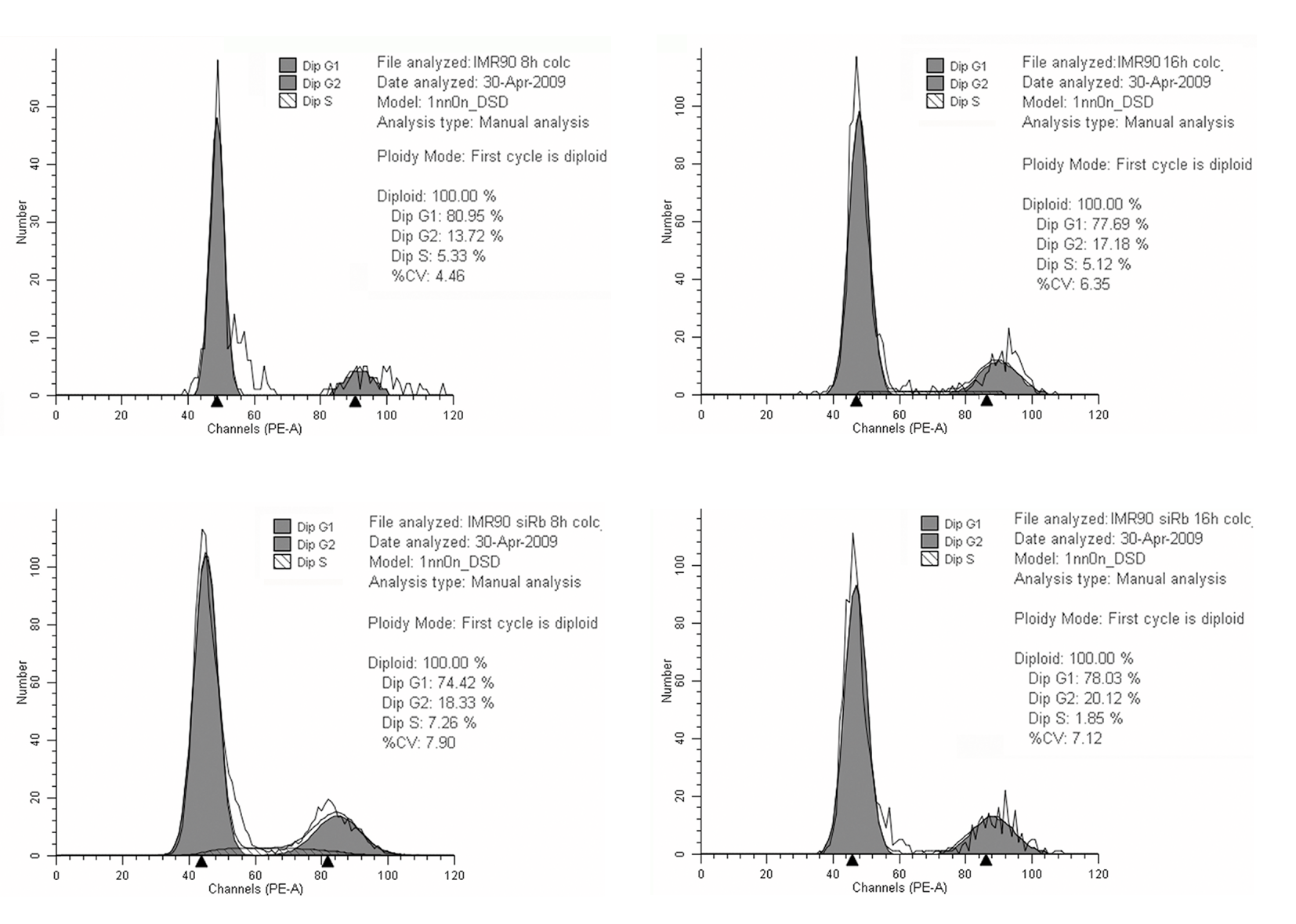

Supplement: Additional file 1 — FACScan analysis of colcemid treated human fibroblasts. Flow citometry analysis showed increase in G2/M of the cells treated with colcemid up to 16 hours. Cell cycle distribution was determined by using FACScanto (Becton Dickinson) and analyzed by ModFit software (Verity). [file 1471-2121-10-79-S1.png]
